# Estimation of the Effect of Soil Texture on Nitrate-Nitrogen Content in Groundwater Using Optical Remote Sensing

**DOI:** 10.3390/ijerph8083416

**Published:** 2011-08-19

**Authors:** Yongyoot Witheetrirong, Nitin Kumar Tripathi, Taravudh Tipdecho, Preeda Parkpian

**Affiliations:** 1 Remote Sensing and GIS Field of Study, School of Engineering and Technology, Asian Institute of Technology, P.O. Box 4, Klong Luang, Pathumthani 12120, Thailand; E-Mails: st103527@ait.ac.th (Y.W.); taravudh@ait.ac.th (T.T.); 2 Environmental Engineering and Management, School of Environment, Resources and Development, Asian Institute of Technology, P.O. Box 4, Klong Luang, Pathumthani 12120, Thailand; E-Mail: preeda@ait.ac.th

**Keywords:** groundwater, spatial autocorrelation, soil texture, Geographic Informationc Systems (GIS), nitrates, remote sensing

## Abstract

The use of chemical fertilizers in Thailand increased exponentially by more than 100-fold from 1961 to 2004. Intensification of agricultural production causes several potential risks to water supplies, especially nitrate-nitrogen (NO_3_^−^-N) pollution. Nitrate is considered a potential pollutant because its excess application can move into streams by runoff and into groundwater by leaching. The nitrate concentration in groundwater increases more than 3-fold times after fertilization and it contaminates groundwater as a result of the application of excess fertilizers for a long time. Soil texture refers to the relative proportion of particles of various sizes in a given soil and it affects the water permeability or percolation rate of a soil. Coarser soils have less retention than finer soils, which in the case of NO_3_^−^-N allows it to leach into groundwater faster, so there is positive relationship between the percentage of sands and NO_3_^−^-N concentration in groundwater wells. This study aimed to estimate the effect of soil texture on NO_3_^−^-N content in groundwater. Optical reflectance data obtained by remote sensing was used in this study. Our hypothesis was that the quantity of nitrogen leached into groundwater through loam was higher than through clay. Nakhon Pathom province, Thailand, was selected as a study area where the terrain is mostly represented by a flat topography. It was found that classified LANDSAT images delineated paddy fields as covering 29.4% of the study area, while sugarcane covered 10.4%, and 60.2% was represented by “others”. The reason for this classified landuse was to determine additional factors, such as vegetation, which might directly affect the quantity of NO_3_^−^-N in soil. Ideally, bare soil would be used as a test site, but in fact, no such places were available in Thailand. This led to an indirect method to estimate NO_3_^−^-N on various soil textures. Through experimentation, it was found that NO_3_^−^-N measured through the loam in sugarcane (I = 0.0054, p < 0.05) was lower than clay represented by paddies (I = 0.0305, p < 0.05). This had a significant negative impact on the assumption. According to the research and local statistical data, farmers have always applied an excess quantity of fertilizer on paddy fields. This is the main reason for the higher quantity of NO_3_^−^-N found in clay than loam in this study. This case might be an exceptional study in terms of quantity of fertilizers applied to agricultural fields.

## 1. Introduction

Existing increasing demands for agricultural products are driven by the growing World population and economic growth. Intensification of agricultural production causes several potential risks to water supplies. Nutrients from chemical fertilizers are more immediately available for plant uptake than in manure, but they may also be more easily leached into groundwater if used in excess [1]. The use of chemical fertilizers in Thailand started to increase exponentially, increasing more than 100-fold from 18,000 tons in 1961 to 2,000,000 tons in 2004, but in spite of this massive increase in chemical fertilizer use, the yields of rice and maize have hardly increased. This suggests a tremendous loss of fertilizers into the environment due to their imbalanced use and poor management [2].

Nitrate-nitrogen (NO_3_^−^-N), which is an essential source of nitrogen (N) for plant growth, is now also considered a potential pollutant by the U.S. Environmental Protection Agency (EPA) [3]. This is because excess applied amounts of NO_3_^−^-N can move into streams by runoff and into groundwater by leaching, thereby becoming an environmental hazard [4]. Many of the major sources of nitrate come from the use and production of fertilizers and waste materials, which are anthropogenic sources of nitrate contamination of groundwater [5]. Although nitrogen exists in many forms, nitrate is the most available form to plants [6]. Nitrate is very soluble in water, and it is readily carried to plant roots as the crop uses water. Soil nitrate unused during the growing season is free to move with water that percolates through the soil. This nitrate has the potential to contaminate groundwater if the water percolates beyond the root zone [7]. NO_3_^−^-N is a problem as a contaminant in drinking water, primarily from groundwater and wells, due to its harmful biological effects [8]. The determining factor in the WHO’s decision to set the Maximum Contaminant Levels (MCLs) at 10 mg/L for the safety limit of drinking water [9], and it was the occurrence of methemoglobinemia in infants under six months [1]. Although the Maximum Contaminant Level for nitrogen was set at 10 mg/L nitrate-nitrogen, in 1976, EPA suggested that water having concentrations above 1 mg/L should not be used for infant feeding [10].

Groundwater in Thailand is a source of household drinking water and supplements surface water for agriculture and livestock uses. Groundwater is used for the public water supply in 20% of the nation’s towns and cities and for half of the sanitary districts. It is estimated that 75% of domestic water is obtained from groundwater sources, and that they serve some 35 million people in villages and in urban areas [1]. The nitrate concentration in the groundwater increased more than three times after fertilization, from 1.8 mg/L to 7.2 mg/L of NO_3_^−^-N, contamination groundwater due to the application of excess fertilizers which have been applied for long time [11].

Soils have varied retentive properties depending on their texture and organic matter content. Soil texture refers to the relative proportion of particles of various sizes in a given soil. Sandy soils have less retention than finer clay soils because sandy soils have less silt and clay [12], which give rise to a lower cation exchange capacity (CEC) [13], in the case of NO_3_^−^-N allows it to leach into groundwater faster, so there is positive relationship between the percentage of sands and NO_3_^−^-N concentration in groundwater wells [14]. CEC is the sum total of exchangeable cations that a soil can absorb. Although NO_3_^−^-N is an anion that can readily leach through the soil profile, soils with significant quantities of silt, clay, and organic matter will also retain more NO_3_^−^-N than soils without much silt and clay. Soil texture also affects water permeability or percolation rate of a soil. Smaller amounts of silt and clay have higher water permeability rates than loamy sands or sandy loams [4].

Remote sensing has the general advantage of providing spatially distributed measurements on a temporal basis. However, it mostly observes the surface of the Earth. There are also below ground remote sensing applications in fields such as geology, hydrogeology, mineralogy, *etc*. Therefore, a link must be established between the surface observation and the subsurface (groundwater) phenomena. Physical features of the landscape such as alignments are detected in satellite images, and these provide valuable information for groundwater investigations [15]. Aerial photography and visible and near-infrared satellite observations are widely used in groundwater exploration [16–18]. Spectral reflectance will be related with nitrate content in groundwater by indirect relationships.

The hypothesis presented herein is the fact that the quantity of nitrate nitrogen accumulated in groundwater through leaching by loam textured soils is higher than that of clay textured ones. This study aimed to estimate the effect of soil texture on NO_3_^−^-N content in groundwater. Optical reflectance data obtained by remote sensing was used in this study.

## 2. Materials and Methods

### 2.1. Data Preparation

**Figure 1.**
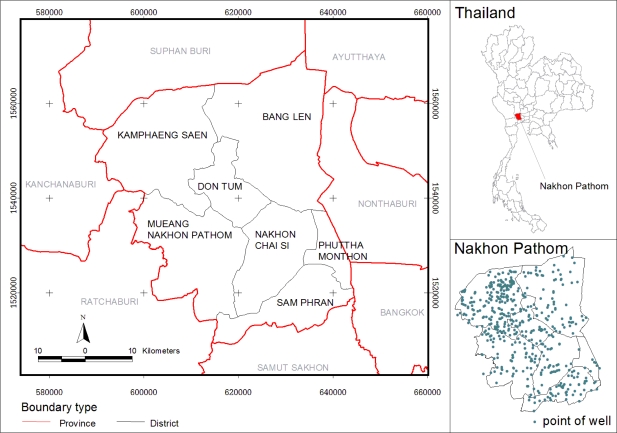
Study area in Nakhon Pathom province, Thailand.

The study site is Nakhon Pathom province in Thailand (Figure 1) which is one of the monitoring provinces for land subsidence of Bangkok and vicinities. The province is divided into seven administrative districts. Most of the area are plains with no mountainous land. Plateaus are found in the west of Amphoe Muang and Amphoe Kamphaeng Saen. The plains are found along the Tha Cheen River. The study covers an area of 2,168 square kilometers, and an area of about 50% of this fertile land is mainly devoted to rice and fruit crops, and 8.16% is sugarcane[19], thus most of the residents earn their living from agriculture.

LANDSAT TM-5 images acquired on 14 May 2010, scene center location (lat/long: 13.917, 100.100), were used to classify for landuse and extract the spectral reflectance. Groundwater analysis data was collected in May 2010 from 32 monitoring stations operated by the Department of Groundwater Resources (DGR). The spatial data layers such as soil unit and geology were provided by the Geo-Informatics and Space Technology Development Agency (GISTDA). The analysis of groundwater properties were derived from secondary data obtained from DGR. Several interpolations were implemented and then the most suitable results was used in the study. Soil texture and soil pH layers were reclassified from soil properties of the top soil layer.

### 2.2. Data Interpolation

Nitrate content (NO_3_^−^-N) from 32 monitor wells were converted to raster data by several interpolation methods in ArcView GIS (extension: Spatial analyst by ESRI and Kriging interpolation), such as IDW, Spline, and Kriging. The most suitable result for this study was selected by accuracy checking from each interpolation method. The extraction of grid value under the point includes checking points were provided. The details of each criterion are Inverse Distance Weighted (IDW) with p = 2; known as the inverse distance squared weighted interpolation, Spline: Weight (W) = 0.1, 0.5; Regular (R), Tension (T), Kriging: Spherical, Circular, Exponential, Gaussian, Linear, Universal1 (linear with linear drift), and Universal2 (linear with quadratic drift).

### 2.3. Spatial Autocorrelation Analysis

The interpolation results were analyzed by Spatial Autocorrelation Analysis. Initially, Moran’s I was used to assess global autocorrelation of the nitrate concentrations for the methods of analysis. Values of Moran’s I less than 0 indicated a negative spatial autocorrelation, *i.e*., clustering of dissimilar values, while those greater than 0 indicated positive spatial clustering, that is, clustering of similar values in similar areas [20]. For the interpolated concentration of NO_3_^−^-N result which is a freeform polygon analysis, a binary weight matrix was created, using Queen Contiguity, identifying which areas were considered neighbors. This method takes into account those areas that share edges to the immediate left, right, up, and down as well as taking diagonal edges into account (reflecting how a queen moves in a game of chess). In this matrix, a “1” was assigned if location *i* was the neighboring location *j*, otherwise a zero was assigned [21]. All spatial autocorrelation analyses were performed using the GeoDa software.

### 2.4. Spectral Extraction Analysis

NO_3_^−^-N that was obtained from Spatial Autocorrelation Analysis was defined as an input for further Spectral Extraction Analysis that incorporated soil texture, soil pH, and landuse. The spectral reflectance related to soil texture, soil pH, and landuse were analyzed.

### 2.5. Statistical Analysis

By interpolation method, statistical analysis (ANOVA and DMRT) were applied to the process of best fit selection based on interpolation technique.

### 2.6. Software

Various software packages, namely ArcView GIS, Spatial analyst by ESRI, Kriging Interpolation Extension 2.01 [22], ENVI, SPSS statistics, and GeoDa, were used in this study.

## 3. Results

### 3.1. Data Preparation

#### 3.1.1. LANDSAT Imagery Data

**Figure 2.**
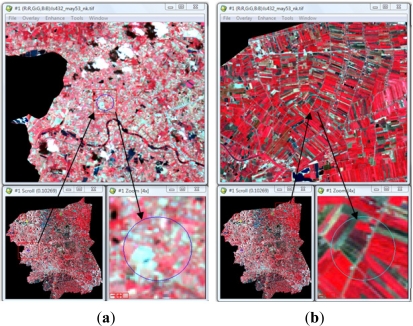
The false color composite of LANDSAT imagery data (432) of Nakhon Pathom, Thailand.

**Figure 3.**
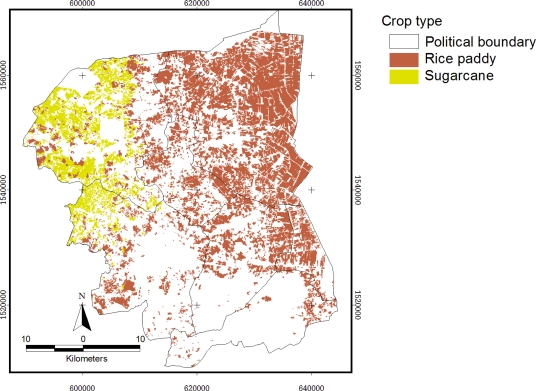
Main landuses of the study area in Nakhon Pathom in 2010.

Visual interpretation was used to classify an agricultural main crop in this study. Figure 2 represents LANDSAT image of the samples’ rice paddy field and sugarcane areas (Figure 3).

#### 3.1.2. Soil Texture

**Figure 4.**
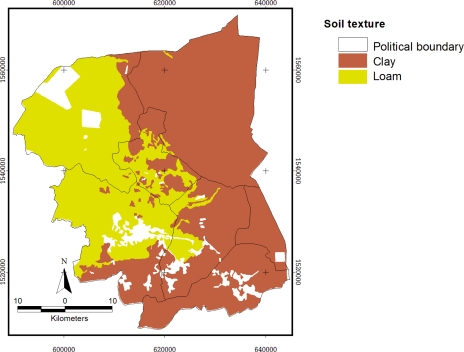
Map of soil texture classes in the study site.

There were only two soil textures: loam (36.7%) and clay (63.3%). Soil texture provides a standard terminology related to the relative percentages of sand, silt, and clay. Texture controls the capacity of the soil to attract and bind potential pollutants to individual soil particles. Finer texture soils have the greater capacity to provide exchange sites and thereby bind pollutants, which result in a lower transport potential. Coarser-textured soils function oppositely in that transport potential is higher and thereby the risk for ground water contamination is relatively greater [23]. Figure 4 shows that most of the topsoil cover in the study site was covered by clay from the North to South, and loam was found in the Northwest; white color (blank area) means an area which is not available in the soil map.

#### 3.1.3. Soil pH

**Figure 5.**
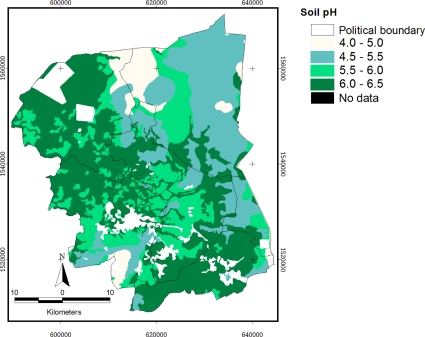
Map of soil pH classes in the study site.

For the study of soil series in Thailand, the Land Development Department (LDD) has classified soils into 62 Great Group phases, focusing on the soil suitability related to crop cultivation [24]. A soil pH map (Figure 5) was generated from the average pH of the Great Group phases in the study area. The classes of soil pH which were reclassified are described in Table 1.

#### 3.1.4. Groundwater Pond

**Figure 6.**
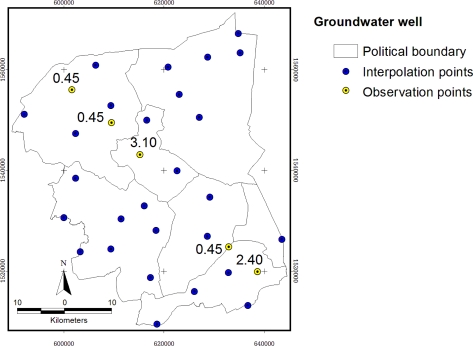
Location of groundwater wells.

The study area includes 32 monitoring groundwater ponds, see Figure 6. The circular area of 500 meters buffered from groundwater ponds represents the sample area of sugarcane (a) and rice paddy (b), the center of each circle is the pond. The randomly selected five points were shown in yellow color with concentration of NO_3_^−^-N in mg/L and the remaining 27 points for interpolation, was shown in blue color.

### 3.2. NO_3_^−^-N Interpolation

**Figure 7.**
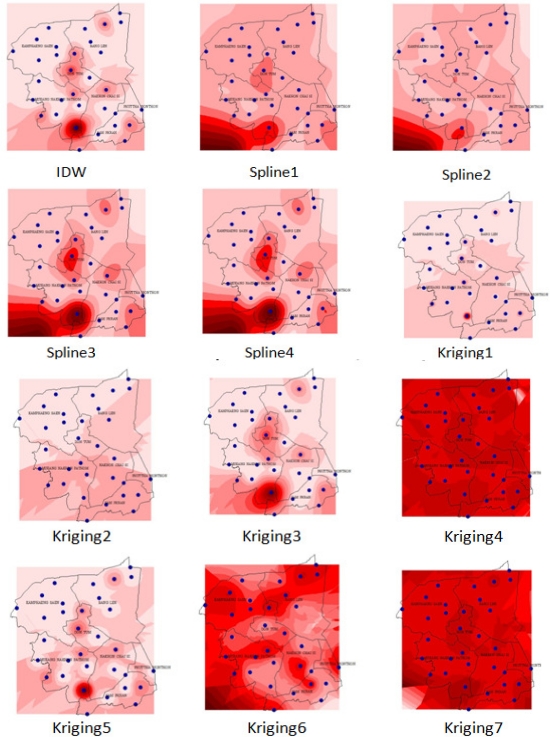
Surface results from several interpolation methods, darker colors mean higher NO_3_^−^-N content in groundwater.

The interpolation method results in Figure 7 show several NO_3_^−^-N patterns in the study site. The fixed effects model analysis of variance (ANOVA) [27,28] was used to figure out the most suitable interpolation method across the study area. In Table 2, the interpolation method experiments of NO_3_^−^-N concentration in Nakhon Pathom province showed that the treatment with KRIG3 of the interpolation methods showed the highest NO_3_^−^-N concentration in groundwater (1.1013), but KRIG6 showed the lowest (1.0002), while the treatment with Sample NO_3_^−^-N is the observed data (1.0969).

**Figure 8.**
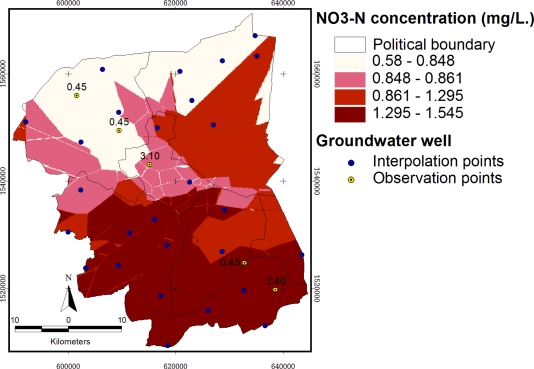
Nitrate interpolated by Kriging employing Gaussian.

The results from Table 2 show that the NO_3_^−^-N concentration mean was not significantly different at the 5% level. The most suitable result, which is the closest mean of an interpolation method to the observed data and KRIG4 as the selected method, was Kriging with Gaussian model (Figure 8). The input data for the Kriging interpolation carried out using a Gaussian model, is NO_3_^−^-N concentration in mg/L, output cell size = 100 × 100 meters, search radius distance = 10,000 meters, and number of neighbors = 12. The limiting distance was defined based on the results of pattern analysis and the minimum requirements of Kriging interpolation.

### 3.3. Spatial Autocorrelation Analysis

**Figure 9.**
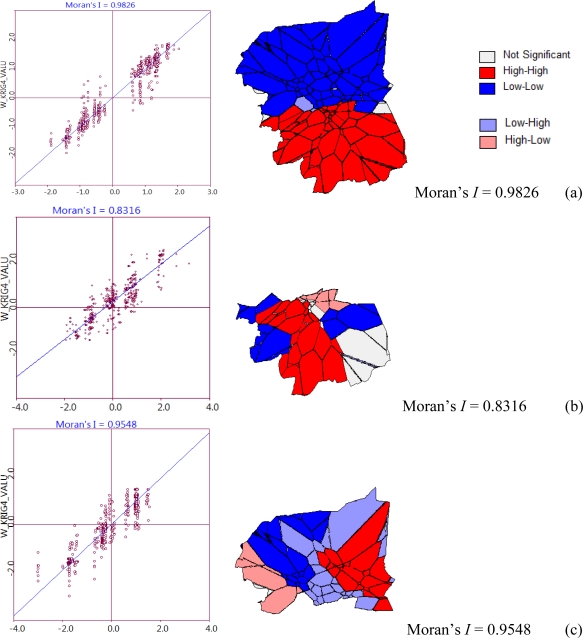
Spatial autocorrelation analysis of NO_3_^−^-N.

To process the spatial statistical analysis, the interpolation result surface from the Kriging with Gaussian criteria process was converted from the grid (100 meter grid size) to polygon data layer. Means of each grid value was generated to be the polygon value by reclassification. The result of this analysis is shown in Figure 9.

Figure 9 shows that the results from an analysis of spatial autocorrelation from Kriging interpolation with Gaussian; (a) the analysis for whole study area, the reclassification of the cluster from High-High value and Low-Low value in (b) and (c), respectively. Classes of nitrate with sub-clusters were shown in small letters, so HH-hh means “high-high cluster which was found in High-High from Figure 9 (b)”, while on the other hand HH-ll means low-low in High-High.

### 3.4. Spectral Extraction Analysis

**Figure 10.**
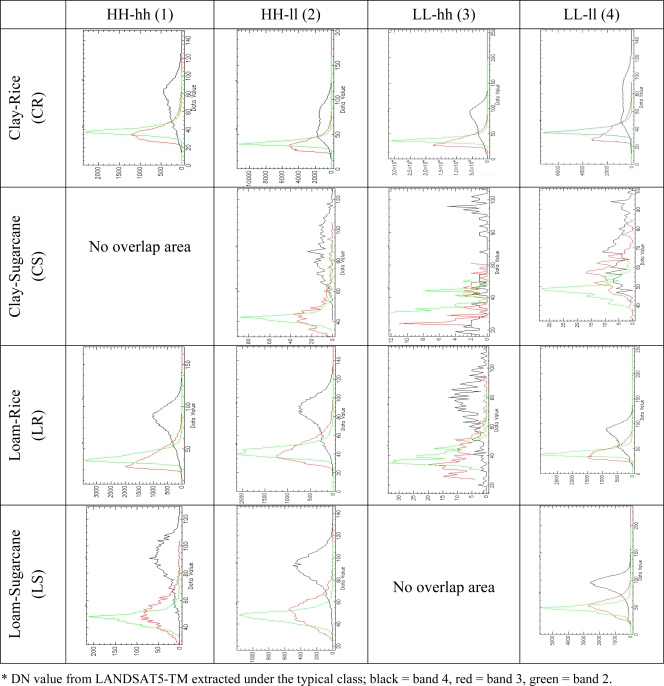
The spectral reflectance value and layer classes.

To investigate the spectral reflectance activities of the ground, digital numbers were extracted using the ENVI software (Environment for Visualizing Images) and the results are shown in Figure 10.

Figure 10 shows that the comparison of the statistical analysis results from an experiment by extracting the spectral reflectance from satellite data under different sampling areas. Chart of an experimental result for study the relation of nitrogen concentration in groundwater and the other layers which display by nitrate-nitrogen in x-axis and average spectral reflectance in y-axis. The result from the extraction is described as follows.

Clay-Rice (CR): The biggest amount of pixels from extraction showed a clustered pattern and covered 14.76% of the provincial area. The 1CR class gave the most uniform curve on the DN value of band 4, meaning a very close stage of planting and land cover, while 2CR, 3CR, and 4CR represented the double peak of value, that shows the different planting stages.

Clay-Sugarcane (CS): This random pattern covered 0.03% of the provincial area that was not suitable for sugarcane planting, due to the clay texture. The curve of the DN value was not overlaid on HH-hh. The rest was also not uniform, which shows that the maximum value of band 4 was represented on 2CS, but the minimum in 3CS.

Loam-Rice (LR): This class covered 3.18% of the provincial area. The 1LR and 2LR classes showed a unique curve of DN value from band 4, but 4LR showed a small peak around the value 50, representing the different stages of planting; the lower peak showed the water cover on the ground. The small amount of 3LR will not describe since the very small cover of area, 0.2 square kilometers.

Loam-Sugarcane (LS): This class covered 2.82% of the provincial area and most of the area was suitable for sugarcane. There was the small amount of DN value in 1LS and 2LS, which was the highest area of NO_3_^−^-N. However, a continuous rising curve of band4was found in 4LS.

## 4. Discussion

The study approach followed spectral analysis of relationships between agricultural crops and nitrate concentrations in ground water by comparing two different spatial soil textures. The mean nitrate concentration of 1.0969 mg/L indicates that there is some human influence on nitrate concentrations in groundwater. Therefore, the results of this study should assist in the determination of significant sources of nitrate, helping in the estimation of fertilization practices to keep the levels within acceptable limits, lower than 1 mg/L.

### 4.1. Data Preparation

#### 4.1.1. Landuse Class

Since most of the land in the study site was located in irrigated areas, an individual crop calendar was present in the variety. The main crops in the study site are rice paddy and sugarcane. As seen from Figure 2, the adjacent crop area represents the different stages of planting in both rice paddy and sugarcane fields. These land uses frequently have nitrogen-based fertilizers applied to improve crop yield. Rice paddies are generally heavily fertilized, with a practical average of 40.63 ton/km^2^ of 16-20-0 and 46-0-0 NPK, and sugarcane fertilizer is applied at a rate of is 46.88 ton/km^2^ [21]. Visual interpretation allows determination of the landuse cover: rice paddy field cover 29.4% (629.88 km^2^) and sugarcane cover 10.4% (224.23 km^2^) of whole study area.

#### 4.1.2. Soil Texture

Although there are nine units of soil covered in the area, the top soil presented only two types of texture. The clay texture covers 1,264.56 km^2^ and loam covers 740.84 km^2^, which are related with the rice paddy and sugarcane.

#### 4.1.3. Soil pH

Most of the area is found to be Class 4, which is the highest range of pH class in this study (pH 6.0–6.5) and it covered 862.49 km^2^, or 39.78 % of the provincial area. The lower pH areas cover 554.83 km^2^, 451.94 km^2^, and 125.32 km^2^ for pH 5.5, 6.0, and 4.5, respectively.

#### 4.1.4. Groundwater Pond

**Figure 11.**
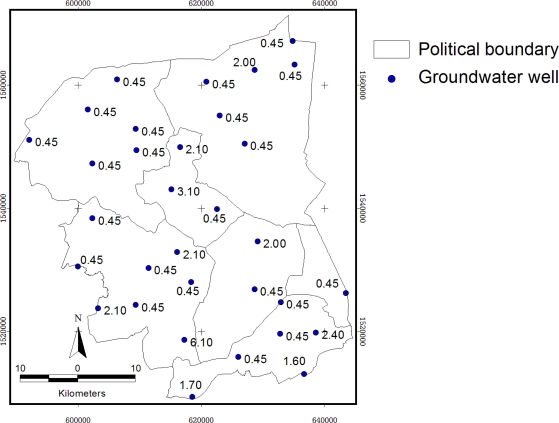
Location of groundwater monitoring stations showing nitrate concentration (mg/L).

Groundwater monitoring stations were distributed in the whole study area (Figure 11). The maximum content of NO_3_^−^-N in groundwater is 6.1 mg/L from the station PD0102, located in the urban area. Mean value is 1.0969, minimum is 0.45 mg/L (less than 0.9 mg/L according to the measurement by ion-selective electrode methods—Department of Groundwater Resources, Bangkok, Thailand, 2009, and the standard deviation is 1.1887.

### 4.2. NO_3_^−^-N Interpolation

The results from Figure 7 show that several methods such as IDW1, spline3, spline4, krig1, krig3, and krig5 have a “bull’s eye effect”. Table 3 shows the observed group of means of the original measured NO_3_^−^-N content from DGR. The comparisons of mean value of nitrate concentrations among interpolation methods were assessed using the fixed-effect models analysis of variance (ANOVA). Each value point was extracted from the grid value under the point, including five check points. There were significant differences in nitrate concentrations among interpolation methods (P > 0.05), as revealed by Duncan’s Multiple Range Test (DMRT). The closest means from their residual when compared with the observed value was selected. Hence, Kriging with Gaussian criteria (KRIG4) was selected to be the most suitable result, as shown in Figure 8.

### 4.3. Spatial Autocorrelation Analysis

The nitrate-nitrogen layer was classified by spatial autocorrelation analysis, which was compared by the mean of local and neighborhood of each other, and then classified into four classes, giving a high local and high neighborhood (HH), high local and low neighborhood (HL), low local and high neighborhood (LL), and low local and high neighborhood (LH). This was represented in the 1st, 2nd, 3rd, and 4th quadrant of the graph, respectively. Spatial autocorrelation analysis of NO_3_^−^-N from Kriging interpolation with Gaussian for whole study area as shown in the Figure 9(a), where the map is clustered into two big paths, up and down. However, the reclassifications of the clusters from separated High-High and Low-Low values in Figures 9(b) and (c) were shown to be more detailed and clustered, with Moran values of 0.8316 and 0.9548, respectively. The local NO_3_^−^-N clusters from HH and LL were divided into four classes of nitrate. Soil pH and soil texture were reclassified into four and two classes, respectively, from a soil unit of the study area from soil map scale 1:100,000 and use only the top layer of each soil unit considering the root zone of the typical crops (30 cm). Landuse class focused on agricultural crop with high nitrate-nitrogen practice which gave two classes of layers. The multiple classes of layer which will be input for the intersection analysis and the number of data layer classes are shown in Table 4.

**Figure 12.**
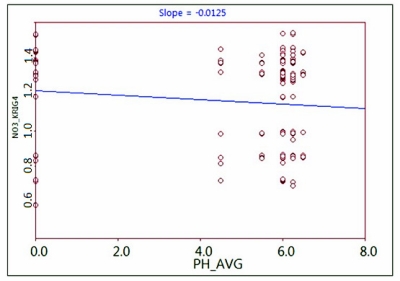
Effects of nitrate concentration in groundwater on the accuracy of the spatial interpolation methods (Kriging-Gaussian) compared in the average soil pH from soil unit.

Although dissolved nitrogen will have the highest concentrations in soil with pH 6–8, the scatter plot combination of NO_3_^−^-N (Kriging-Gaussian) and soil pH (Figure 12), had no significant correlation between soil pH and NO_3_^−^-N content in groundwater (correlation slope = −0.0125). Hence, the soil pH class was not implemented in the intersection and spectral extraction process.

The average of difference method was used to compare an average of nitrate concentration from five points with the observed value. Kriging interpolation provided good results for all criteria, but the most suitable method was selected by the minimum difference from mean of an observed value.

**Figure 13.**
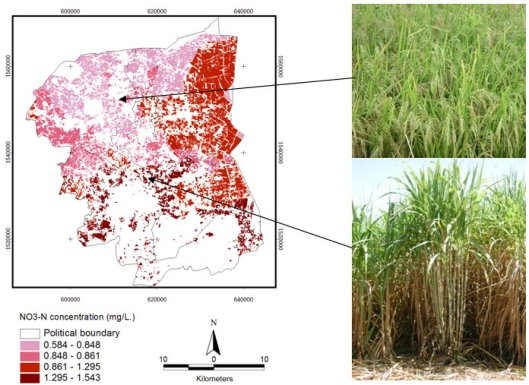
Combination of landuse crops and NO_3_^−^-N concentration from Kiginging-Gaussian interpolation.

The experiment of the combination of NO_3_^−^-N and landuse crops as shown in Figure 13, where high nitrate-nitrogen content in groundwater of the study area were mostly found in the south to east of the study area, which is related to soil texture and landuse crops. However, the National Statistical Office has reported an increasing trend of fertilizer use in Thailand [29]. The use of nitrogen fertilizer (16-20-0 and 46-0-0, NPK) in the study area is also very high. The spectral reflectance extraction was processed from LANDSAT imagery data, which was separated into difference groups by the combination of NO_3_^−^-N data with soil texture and crops.

On the lower right photo is sugarcane, and upper right shows a rice paddy photo dated 20 May 2010. The black point of each crop is the point where the photo was shot and the color gradient represents the concentration of NO_3_^−^-N from groundwater, interpolated from KRIG4, processed by the spatial overlay with landuse crops (rice paddy and sugarcane).

### 4.4. Spectral Extraction Analysis

**Figure 14.**
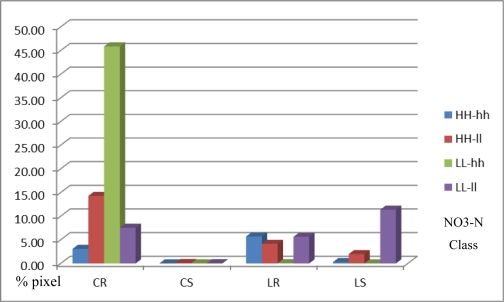
Pixel distribution from spectral extraction analysis.

Results of the extraction were processed in the ENVI software. Several layers were analyzed by spatial overlay analysis. The statistical information of each class is shown in Table 5. The total number of pixels of the LANDSAT imagery is 5,487,093 points; it was clipped with the provincial boundary, and the number of all pixels which were included in this extraction was 721,141 points. There are fourteen spectral results from the extraction, no overlay was present on Clay-Sugarcane/HH-hh (1CS) and Loam-Sugarcane/LL-hh (3LS), so those two results were cut off. The summary of the point numbers is shown in Figure 14.

The contrast comparison of the DN curve from Landsat band 4 found that the reflectance value from the rice paddy field which located on the high NO_3_^−^-N path (HH-hh) representing the unique and dominant peak of the curve, but in the low NO_3_^−^-N path was representing the twice peak of the curve. For the sugarcane field, there was small covering area of sugarcane for high path but on the other hand the low path still represents the unique peak in the lowest path of NO_3_^−^-N concentration (4LS). However, the study found some irregular pattern of the curve in the classes of 2CS, 3CS, 3LR, and 4CS.

## 5. Conclusions

The nitrate was classified into four classes by spatial autocorrelation analysis (Moran’s I and Local Moran statistics; LISA), by a means comparison. The cluster map legend contains five categories: Not significant (Areas that are not significant at a default pseudo significance level of 0.05), High-High (High values surrounded by high values), Low-Low (Low values surrounded by low values), Low-High (Low values surrounded by high values), and High-Low (High values surrounded by low values). There were 2 classes of the global clustered of NO_3_^−^-N with values of *Moran’s I* of 0.8316 (p = 0.05) for HH, and I = 0.9548 (p = 0.01). However, the local Moran’s statistics shown HH-hh (I = 0.8182, p < 0.01), HH-ll (I = 0.3486, p < 0.01), LL-hh (I = 0.6534, p = 0.01), and LL-LL (I = 0.4065, p < 0.5). The effect of soil texture on nitrate-nitrogen content in groundwater was directly observed by its reflectance values through remote sensing. It was found that NO_3_^−^-N measured through the loam in sugarcane (I = 0.0054, p < 0.05) was lower than clay represented in paddy (I = 0.0305, p < 0.05). This had a significant negative impact on the assumption, the quantity of nitrogen leached into groundwater through loam was higher than through clay.

According to the research [2] and local statistical data [19], farmers always apply excess fertilizer to paddy fields. This is a main reason for the higher quantity of NO_3_^−^-N found in clay than in loam in this study. This case might be an exceptional study in terms of the quantity of fertilizers applied to agricultural fields. There was high level of NO_3_^−^-N contaminants in urban areas, showing that there are other sources of contaminants. Therefore, there is a need to investigate the combined and multiple sources of contamination in urban areas that can cause hazard to urban populations.

## Figures and Tables

**Table 1.** The classes of soil pH reclassified by several soil series.

**Class**	**pH range**	Group of soil series *	**Min**	**Max**	**Average**	**Area (Km^2^)**

1	4.0–5.0	11, 11f	4.0	5.0	4.5	125.32
2	5.0–5.5	2, 2f, 2/11	5.0	6.0	5.5	554.83
3	5.5–6.0	1, 1/2, 1f, 4, 4/38	5.5	6.5	6.0	451.94
4	6.0–6.5	3, 3f, 8, 8/2, 8/3, 38, 38/7, 38B	5.5	7.0	6.25	383.22
		7, 33, 33/7	6.0	7.0	6.5	479.27

* Group of soil series in Thailand [24–26].

**Table 2.** Average concentration of NO_3_^−^-N varieties in interpolation method.

**Interpolation Methods**	**NO_3_^−^-N concentration in groundwater (mg/L)**

Observed (Sample NO_3_^−^-N)	1.0969
IDW1	1.0100
SPLINE1	1.0577
SPLINE2	1.0607
SPLINE3	1.0205
SPLINE4	1.0205
KRIG1	1.1030
KRIG2	1.1016
KRIG3	1.1032
KRIG4	1.1013
KRIG5	1.0505
KRIG6	1.0002
KRIG7	1.0065
F-test	not significant
CV (%)	87.2370

**Table 3.** The comparison of NO_3_^−^-N interpolation methods by mean of NO_3_^−^-N concentration (mg/L).

**Treatment**	**Mean (NO_3_^−^-N in mg/L)**	Δ Observe *

Observed (Sample NO_3_^−^-N)	**1.0969**	**0.0000**
IDW1	1.0100	0.0869
SPLINE1	1.0577	0.0392
SPLINE2	1.0607	0.0362
SPLINE3	1.0205	0.0764
SPLINE4	1.0205	0.0764
KRIG1	1.1030	0.0061
KRIG2	1.1016	0.0047
KRIG3	1.1032	0.0063
**KRIG4**	**1.1013**	**0.0044**
KRIG5	1.0505	0.0464
KRIG6	1.0002	0.0967
KRIG7	1.0065	0.0904

* Calculated from the absolute difference of the observed value and each method.

**Table 4.** Layer classes of data in this study.

**Layer**	**Class 1**	**Class 2**	**Class 3**	**Class 4**

Nitrate	HH-hh	HH-ll	LL-hh	LL-ll
Soil pH	4.5	5.5	6.0	6.5
Soil Texture	Loam	Clay		
Landuse	Sugarcane	Paddy field		

**Table 5.** Statistical information of classes from spatial overlay analysis.

**No.**	**Class**	**NO_3_^−^-N (mg/L) from Kriging-Gaussian**				**Area (%)**

		**Min**	**Max**	**Mean**	**SD**	

1	Study area	0.58	1.54	1.07	0.26	100.00
	
2	1CR	1.30	1.54	1.44	0.06	0.63
3	1CS	No overlap area				0.00
4	1LR	1.25	1.47	1.40	0.05	1.17
5	1LS	1.26	1.45	1.35	0.05	0.05
6	2CR	1.27	1.38	1.27	0.00	2.98
7	2CS	1.25	1.31	1.31	0.01	0.02
8	2LR	1.18	1.37	1.28	0.05	0.84
9	2LS	1.18	1.32	1.24	0.06	0.40
10	3CR	0.98	0.98	0.98	0.00	9.58
11	3CS	0.98	0.98	0.98	0.00	0.00
12	3LR	0.98	0.98	0.98	0.00	0.01
13	3LS	No overlap area				0.00
14	4CR	0.70	1.46	0.79	0.21	1.57
15	4CS	0.69	0.73	0.72	0.01	0.01
16	4LR	0.69	0.86	0.72	0.01	1.16
17	4LS	0.69	1.31	0.79	0.20	2.36

Total						20.79
